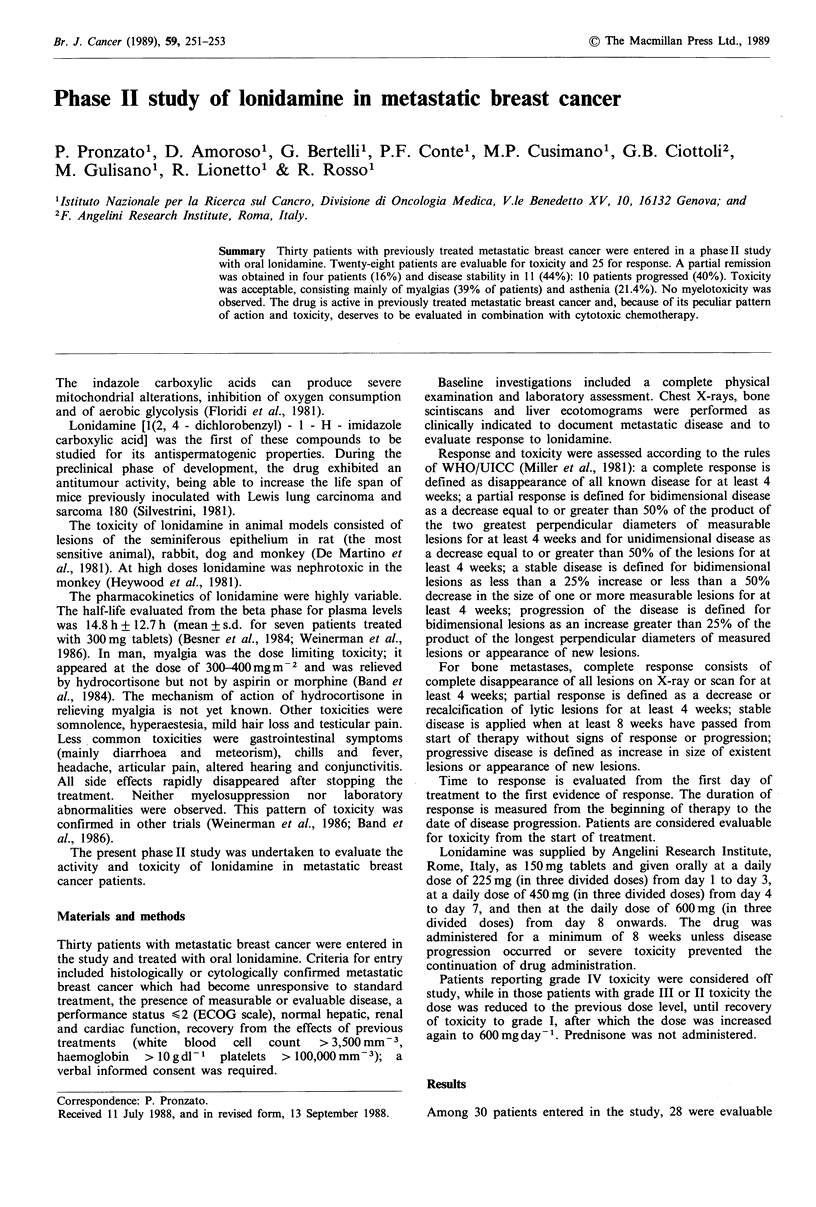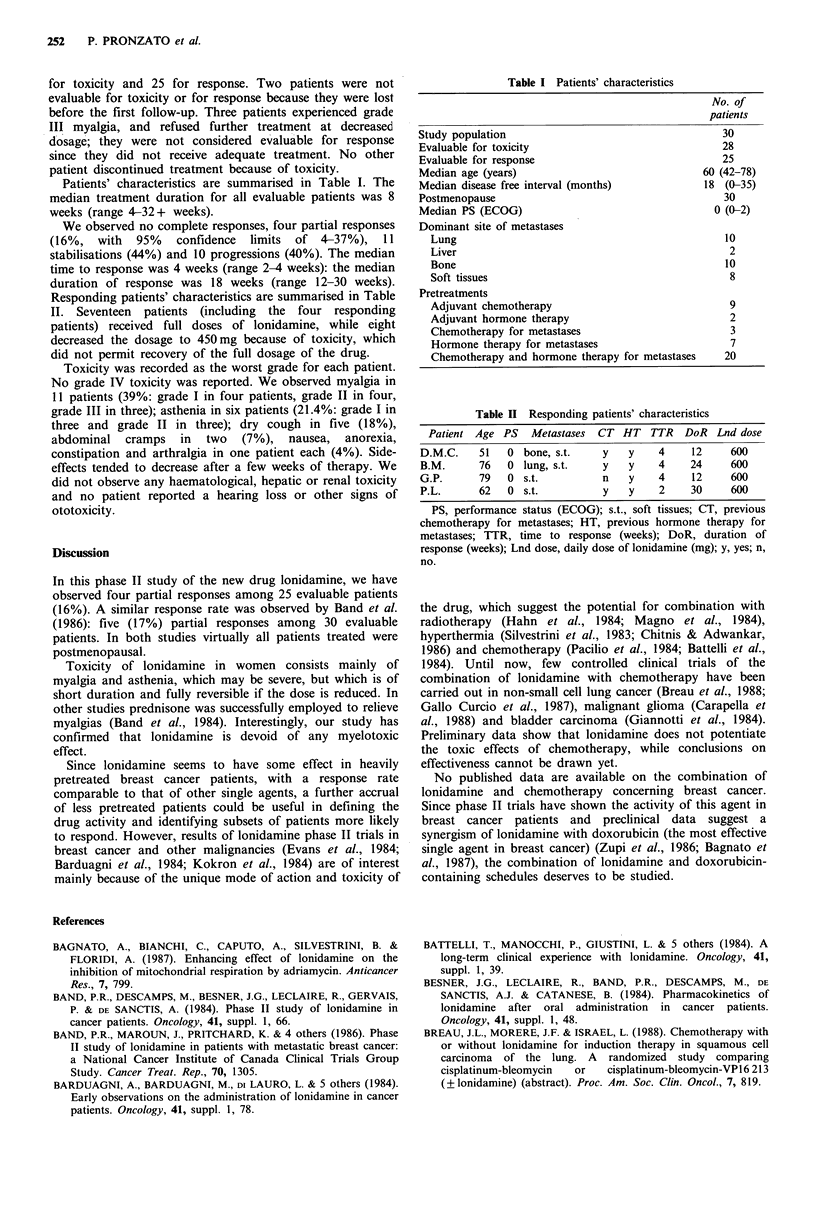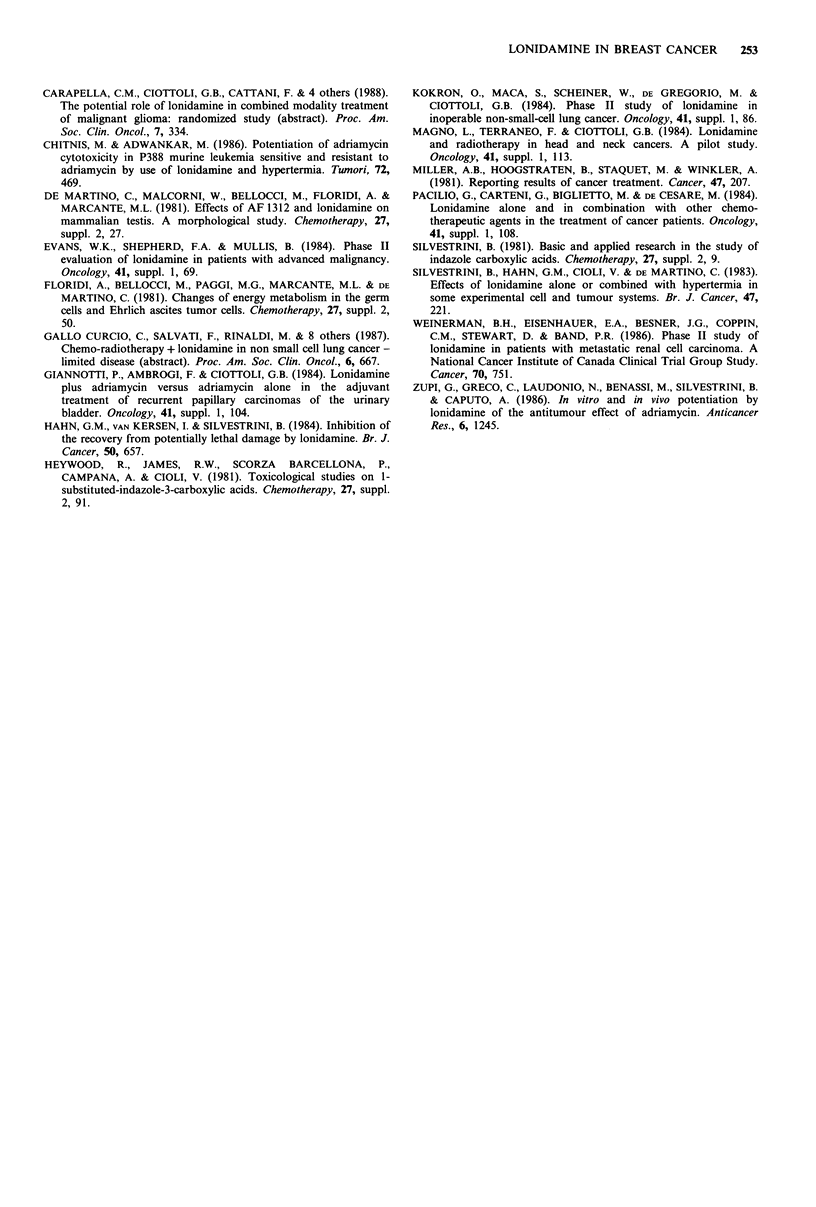# Phase II study of lonidamine in metastatic breast cancer.

**DOI:** 10.1038/bjc.1989.51

**Published:** 1989-02

**Authors:** P. Pronzato, D. Amoroso, G. Bertelli, P. F. Conte, M. P. Cusimano, G. B. Ciottoli, M. Gulisano, R. Lionetto, R. Rosso

**Affiliations:** Istituto Nazionale per la Ricerca sul Cancro, Divisione di Oncologia Medica, Genova, Italy.

## Abstract

Thirty patients with previously treated metastatic breast cancer were entered in a phase II study with oral lonidamine. Twenty-eight patients are evaluable for toxicity and 25 for response. A partial remission was obtained in four patients (16%) and disease stability in 11 (44%): 10 patients progressed (40%). Toxicity was acceptable, consisting mainly of myalgias (39% of patients) and asthenia (21.4%). No myelotoxicity was observed. The drug is active in previously treated metastatic breast cancer and, because of its peculiar pattern of action and toxicity, deserves to be evaluated in combination with cytotoxic chemotherapy.


*Br. J. Cancer* (1989), **59**, 251–253 © The Macmillan Press Ltd., 1989

### Phase II study of lonidamine in metastatic breast cancer

P. Pronzato[1], D. Amoroso[1], G. Bertelli[1], P.F. Conte[1], M.P. Cusimano[1], G.B. Ciottoli[2], M. Gulisano[1], R. Lionetto[1] & R. Rosso[1]

[1]*Istituto Nazionale per la Ricerca sul Cancro, Divisione di Oncologia Medica, V.le Benedetto XV, 10, 16132 Genova; and* [2]*F. Angelini Research Institute, Roma, Italy.*

**Summary** Thirty patients with previously treated metastatic breast cancer were entered in a phase II study with oral lonidamine. Twenty-eight patients are evaluable for toxicity and 25 for response. A partial remission was obtained in four patients (16%) and disease stability in 11 (44%): 10 patients progressed (40%). Toxicity was acceptable, consisting mainly of myalgias (39% of patients) and asthenia (21.4%). No myelotoxicity was observed. The drug is active in previously treated metastatic breast cancer and, because of its peculiar pattern of action and toxicity, deserves to be evaluated in combination with cytotoxic chemotherapy.

The indazole carboxylic acids can produce severe mitochondrial alterations, inhibition of oxygen consumption and of aerobic glycolysis (Floridi *et al.*, 1981).

Lonidamine [1(2, 4 - dichlorobenzyl) - 1 - H - imidazole carboxylic acid] was the first of these compounds to be studied for its antispermatogenic properties. During the preclinical phase of development, the drug exhibited an antitumour activity, being able to increase the life span of mice previously inoculated with Lewis lung carcinoma and sarcoma 180 (Silvestrini, 1981).

The toxicity of lonidamine in animal models consisted of lesions of the seminiferous epithelium in rat (the most sensitive animal), rabbit, dog and monkey (De Martino *et al.*, 1981). At high doses lonidamine was nephrotoxic in the monkey (Heywood *et al.*, 1981).

The pharmacokinetics of lonidamine were highly variable. The half-life evaluated from the beta phase for plasma levels was $14.8\,h \pm 12.7\,h$ (mean ± s.d. for seven patients treated with 300 mg tablets) (Besner *et al.*, 1984; Weinerman *et al.*, 1986). In man, myalgia was the dose limiting toxicity; it appeared at the dose of 300–400 $mg\,m^{-2}$ and was relieved by hydrocortisone but not by aspirin or morphine (Band *et al.*, 1984). The mechanism of action of hydrocortisone in relieving myalgia is not yet known. Other toxicities were somnolence, hyperaestesia, mild hair loss and testicular pain. Less common toxicities were gastrointestinal symptoms (mainly diarrhoea and meteorism), chills and fever, headache, articular pain, altered hearing and conjunctivitis. All side effects rapidly disappeared after stopping the treatment. Neither myelosuppression nor laboratory abnormalities were observed. This pattern of toxicity was confirmed in other trials (Weinerman *et al.*, 1986; Band *et al.*, 1986).

The present phase II study was undertaken to evaluate the activity and toxicity of lonidamine in metastatic breast cancer patients.

### Materials and methods

Thirty patients with metastatic breast cancer were entered in the study and treated with oral lonidamine. Criteria for entry included histologically or cytologically confirmed metastatic breast cancer which had become unresponsive to standard treatment, the presence of measurable or evaluable disease, a performance status $\leqslant 2$ (ECOG scale), normal hepatic, renal and cardiac function, recovery from the effects of previous treatments (white blood cell count $>3{,}500\,mm^{-3}$, haemoglobin $>10\,g\,dl^{-1}$ platelets $>100{,}000\,mm^{-3}$); a verbal informed consent was required.

Baseline investigations included a complete physical examination and laboratory assessment. Chest X-rays, bone scintiscans and liver ecotomograms were performed as clinically indicated to document metastatic disease and to evaluate response to lonidamine.

Response and toxicity were assessed according to the rules of WHO/UICC (Miller *et al.*, 1981): a complete response is defined as disappearance of all known disease for at least 4 weeks; a partial response is defined for bidimensional disease as a decrease equal to or greater than 50% of the product of the two greatest perpendicular diameters of measurable lesions for at least 4 weeks and for unidimensional disease as a decrease equal to or greater than 50% of the lesions for at least 4 weeks; a stable disease is defined for bidimensional lesions as less than a 25% increase or less than a 50% decrease in the size of one or more measurable lesions for at least 4 weeks; progression of the disease is defined for bidimensional lesions as an increase greater than 25% of the product of the longest perpendicular diameters of measured lesions or appearance of new lesions.

For bone metastases, complete response consists of complete disappearance of all lesions on X-ray or scan for at least 4 weeks; partial response is defined as a decrease or recalcification of lytic lesions for at least 4 weeks; stable disease is applied when at least 8 weeks have passed from start of therapy without signs of response or progression; progressive disease is defined as increase in size of existent lesions or appearance of new lesions.

Time to response is evaluated from the first day of treatment to the first evidence of response. The duration of response is measured from the beginning of therapy to the date of disease progression. Patients are considered evaluable for toxicity from the start of treatment.

Lonidamine was supplied by Angelini Research Institute, Rome, Italy, as 150 mg tablets and given orally at a daily dose of 225 mg (in three divided doses) from day 1 to day 3, at a daily dose of 450 mg (in three divided doses) from day 4 to day 7, and then at the daily dose of 600 mg (in three divided doses) from day 8 onwards. The drug was administered for a minimum of 8 weeks unless disease progression occurred or severe toxicity prevented the continuation of drug administration.

Patients reporting grade IV toxicity were considered off study, while in those patients with grade III or II toxicity the dose was reduced to the previous dose level, until recovery of toxicity to grade I, after which the dose was increased again to 600 $mg\,day^{-1}$. Prednisone was not administered.

### Results

Among 30 patients entered in the study, 28 were evaluable

Correspondence: P. Pronzato.
Received 11 July 1988, and in revised form, 13 September 1988.

for toxicity and 25 for response. Two patients were not evaluable for toxicity or for response because they were lost before the first follow-up. Three patients experienced grade III myalgia, and refused further treatment at decreased dosage; they were not considered evaluable for response since they did not receive adequate treatment. No other patient discontinued treatment because of toxicity.

Patients' characteristics are summarised in Table I. The median treatment duration for all evaluable patients was 8 weeks (range 4–32+ weeks).

We observed no complete responses, four partial responses (16%, with 95% confidence limits of 4–37%), 11 stabilisations (44%) and 10 progressions (40%). The median time to response was 4 weeks (range 2–4 weeks): the median duration of response was 18 weeks (range 12–30 weeks). Responding patients' characteristics are summarised in Table II. Seventeen patients (including the four responding patients) received full doses of lonidamine, while eight decreased the dosage to 450 mg because of toxicity, which did not permit recovery of the full dosage of the drug.

Toxicity was recorded as the worst grade for each patient. No grade IV toxicity was reported. We observed myalgia in 11 patients (39%: grade I in four patients, grade II in four, grade III in three); asthenia in six patients (21.4%: grade I in three and grade II in three); dry cough in five (18%), abdominal cramps in two (7%), nausea, anorexia, constipation and arthralgia in one patient each (4%). Side-effects tended to decrease after a few weeks of therapy. We did not observe any haematological, hepatic or renal toxicity and no patient reported a hearing loss or other signs of ototoxicity.

**Table I** Patients' characteristics

| | *No. of patients* |
|---|---|
| Study population | 30 |
| Evaluable for toxicity | 28 |
| Evaluable for response | 25 |
| Median age (years) | 60 (42–78) |
| Median disease free interval (months) | 18 (0–35) |
| Postmenopause | 30 |
| Median PS (ECOG) | 0 (0–2) |
| Dominant site of metastases | |
| Lung | 10 |
| Liver | 2 |
| Bone | 10 |
| Soft tissues | 8 |
| Pretreatments | |
| Adjuvant chemotherapy | 9 |
| Adjuvant hormone therapy | 2 |
| Chemotherapy for metastases | 3 |
| Hormone therapy for metastases | 7 |
| Chemotherapy and hormone therapy for metastases | 20 |

**Table II** Responding patients' characteristics

| *Patient* | *Age* | *PS* | *Metastases* | *CT* | *HT* | *TTR* | *DoR* | *Lnd dose* |
|---|---|---|---|---|---|---|---|---|
| D.M.C. | 51 | 0 | bone, s.t. | y | y | 4 | 12 | 600 |
| B.M. | 76 | 0 | lung, s.t. | y | y | 4 | 24 | 600 |
| G.P. | 79 | 0 | s.t. | n | y | 4 | 12 | 600 |
| P.L. | 62 | 0 | s.t. | y | y | 2 | 30 | 600 |

PS, performance status (ECOG); s.t., soft tissues; CT, previous chemotherapy for metastases; HT, previous hormone therapy for metastases; TTR, time to response (weeks); DoR, duration of response (weeks); Lnd dose, daily dose of lonidamine (mg); y, yes; n, no.

### Discussion

In this phase II study of the new drug lonidamine, we have observed four partial responses among 25 evaluable patients (16%). A similar response rate was observed by Band *et al.* (1986): five (17%) partial responses among 30 evaluable patients. In both studies virtually all patients treated were postmenopausal.

Toxicity of lonidamine in women consists mainly of myalgia and asthenia, which may be severe, but which is of short duration and fully reversible if the dose is reduced. In other studies prednisone was successfully employed to relieve myalgias (Band *et al.*, 1984). Interestingly, our study has confirmed that lonidamine is devoid of any myelotoxic effect.

Since lonidamine seems to have some effect in heavily pretreated breast cancer patients, with a response rate comparable to that of other single agents, a further accrual of less pretreated patients could be useful in defining the drug activity and identifying subsets of patients more likely to respond. However, results of lonidamine phase II trials in breast cancer and other malignancies (Evans *et al.*, 1984; Barduagni *et al.*, 1984; Kokron *et al.*, 1984) are of interest mainly because of the unique mode of action and toxicity of the drug, which suggest the potential for combination with radiotherapy (Hahn *et al.*, 1984; Magno *et al.*, 1984), hyperthermia (Silvestrini *et al.*, 1983; Chitnis & Adwankar, 1986) and chemotherapy (Pacilio *et al.*, 1984; Battelli *et al.*, 1984). Until now, few controlled clinical trials of the combination of lonidamine with chemotherapy have been carried out in non-small cell lung cancer (Breau *et al.*, 1988; Gallo Curcio *et al.*, 1987), malignant glioma (Carapella *et al.*, 1988) and bladder carcinoma (Giannotti *et al.*, 1984). Preliminary data show that lonidamine does not potentiate the toxic effects of chemotherapy, while conclusions on effectiveness cannot be drawn yet.

No published data are available on the combination of lonidamine and chemotherapy concerning breast cancer. Since phase II trials have shown the activity of this agent in breast cancer patients and preclinical data suggest a synergism of lonidamine with doxorubicin (the most effective single agent in breast cancer) (Zupi *et al.*, 1986; Bagnato *et al.*, 1987), the combination of lonidamine and doxorubicin-containing schedules deserves to be studied.

### References

BAGNATO, A., BIANCHI, C., CAPUTO, A., SILVESTRINI, B. & FLORIDI, A. (1987). Enhancing effect of lonidamine on the inhibition of mitochondrial respiration by adriamycin. *Anticancer Res.*, **7**, 799.

BAND, P.R., DESCAMPS, M., BESNER, J.G., LECLAIRE, R., GERVAIS, P. & DE SANCTIS, A. (1984). Phase II study of lonidamine in cancer patients. *Oncology*, **41**, suppl. 1, 66.

BAND, P.R., MAROUN, J., PRITCHARD, K. & 4 others (1986). Phase II study of lonidamine in patients with metastatic breast cancer: a National Cancer Institute of Canada Clinical Trials Group Study. *Cancer Treat. Rep.*, **70**, 1305.

BARDUAGNI, A., BARDUAGNI, M., DI LAURO, L. & 5 others (1984). Early observations on the administration of lonidamine in cancer patients. *Oncology*, **41**, suppl. 1, 78.

BATTELLI, T., MANOCCHI, P., GIUSTINI, L. & 5 others (1984). A long-term clinical experience with lonidamine. *Oncology*, **41**, suppl. 1, 39.

BESNER, J.G., LECLAIRE, R., BAND, P.R., DESCAMPS, M., DE SANCTIS, A.J. & CATANESE, B. (1984). Pharmacokinetics of lonidamine after oral administration in cancer patients. *Oncology*, **41**, suppl. 1, 48.

BREAU, J.L., MORERE, J.F. & ISRAEL, L. (1988). Chemotherapy with or without lonidamine for induction therapy in squamous cell carcinoma of the lung. A randomized study comparing cisplatinum-bleomycin or cisplatinum-bleomycin-VP16 213 (± lonidamine) (abstract). *Proc. Am. Soc. Clin. Oncol.*, **7**, 819.

CARAPELLA, C.M., CIOTTOLI, G.B., CATTANI, F. & 4 others (1988). The potential role of lonidamine in combined modality treatment of malignant glioma: randomized study (abstract). *Proc. Am. Soc. Clin. Oncol.*, **7**, 334.

CHITNIS, M. & ADWANKAR, M. (1986). Potentiation of adriamycin cytotoxicity in P388 murine leukemia sensitive and resistant to adriamycin by use of lonidamine and hypertermia. *Tumori,* **72,** 469.

DE MARTINO, C., MALCORNI, W., BELLOCCI, M., FLORIDI, A. & MARCANTE, M.L. (1981). Effects of AF 1312 and lonidamine on mammalian testis. A morphological study. *Chemotherapy*, **27,** suppl. 2, 27.

EVANS, W.K., SHEPHERD, F.A. & MULLIS, B. (1984). Phase II evaluation of lonidamine in patients with advanced malignancy. *Oncology*, **41,** suppl. 1, 69.

FLORIDI, A., BELLOCCI, M., PAGGI, M.G., MARCANTE, M.L. & DE MARTINO, C. (1981). Changes of energy metabolism in the germ cells and Ehrlich ascites tumor cells. *Chemotherapy*, **27,** suppl. 2, 50.

GALLO CURCIO, C., SALVATI, F., RINALDI, M. & 8 others (1987). Chemo-radiotherapy + lonidamine in non small cell lung cancer – limited disease (abstract). *Proc. Am. Soc. Clin. Oncol.*, **6,** 667.

GIANNOTTI, P., AMBROGI, F. & CIOTTOLI, G.B. (1984). Lonidamine plus adriamycin versus adriamycin alone in the adjuvant treatment of recurrent papillary carcinomas of the urinary bladder. *Oncology*, **41,** suppl. 1, 104.

HAHN, G.M., VAN KERSEN, I. & SILVESTRINI, B. (1984). Inhibition of the recovery from potentially lethal damage by lonidamine. *Br. J. Cancer*, **50,** 657.

HEYWOOD, R., JAMES, R.W., SCORZA BARCELLONA, P., CAMPANA, A. & CIOLI, V. (1981). Toxicological studies on 1-substituted-indazole-3-carboxylic acids. *Chemotherapy*, **27,** suppl. 2, 91.

KOKRON, O., MACA, S., SCHEINER, W., DE GREGORIO, M. & CIOTTOLI, G.B. (1984). Phase II study of lonidamine in inoperable non-small-cell lung cancer. *Oncology*, **41,** suppl. 1, 86.

MAGNO, L., TERRANEO, F. & CIOTTOLI, G.B. (1984). Lonidamine and radiotherapy in head and neck cancers. A pilot study. *Oncology*, **41,** suppl. 1, 113.

MILLER, A.B., HOOGSTRATEN, B., STAQUET, M. & WINKLER, A. (1981). Reporting results of cancer treatment. *Cancer*, **47,** 207.

PACILIO, G., CARTENI, G., BIGLIETTO, M. & DE CESARE, M. (1984). Lonidamine alone and in combination with other chemotherapeutic agents in the treatment of cancer patients. *Oncology*, **41,** suppl. 1, 108.

SILVESTRINI, B. (1981). Basic and applied research in the study of indazole carboxylic acids. *Chemotherapy*, **27,** suppl. 2, 9.

SILVESTRINI, B., HAHN, G.M., CIOLI, V. & DE MARTINO, C. (1983). Effects of lonidamine alone or combined with hypertermia in some experimental cell and tumour systems. *Br. J. Cancer*, **47,** 221.

WEINERMAN, B.H., EISENHAUER, E.A., BESNER, J.G., COPPIN, C.M., STEWART, D. & BAND, P.R. (1986). Phase II study of lonidamine in patients with metastatic renal cell carcinoma. A National Cancer Institute of Canada Clinical Trial Group Study. *Cancer*, **70,** 751.

ZUPI, G., GRECO, C., LAUDONIO, N., BENASSI, M., SILVESTRINI, B. & CAPUTO, A. (1986). *In vitro* and *in vivo* potentiation by lonidamine of the antitumour effect of adriamycin. *Anticancer Res.*, **6,** 1245.